# Soft Polymers for Building up Small and Smallest Blood Supplying Systems by Stereolithography

**DOI:** 10.3390/jfb3020257

**Published:** 2012-03-29

**Authors:** Wolfdietrich Meyer, Sascha Engelhardt, Esther Novosel, Burkhard Elling, Michael Wegener, Hartmut Krüger

**Affiliations:** 1 Fraunhofer Institute of Applied Polymer Research (IAP), Geiselbergstr 69, Potsdam 14476, Germany; Email: burkhard.elling@iap.fraunhofer.de (B.E.); michael.wegener@iap.fraunhofer.de (M.W.); hartmut.krueger@iap.fraunhofer.de (H.K.); 2 Rheinisch-Westfälische Technische Hochschule Aachen, RWTH Aachen, Steinbachstraße 15, Aachen 52074, Germany; Email: sascha.engelhardt@ilt.fraunhofer.de; 3 Fraunhofer Institute for Interfacial Engineering and Biotechnology (IGB), Nobelstraße 12, Stuttgart 70569, Germany; Email: esther.novosel@igb.fraunhofer.de

**Keywords:** functional biopolymers, biocompatibility, biomedical devices, tissue devices, rapid prototyping

## Abstract

Synthesis of a homologous series of photo-polymerizable α,ω-polytetrahydrofuranether-diacrylate (PTHF-DA) resins is described with characterization by NMR, GPC, DSC, soaking and rheometrical measurements. The curing speeds of the resins are determined under UV light exposure. Young’s modulus and tensile strength of fully cured resins show flexible to soft material attributes dependent on the molar mass of the used linear PTHF-diacrylates. Structuring the materials by stereo lithography (SL) and multiphoton polymerization (MPP) leads to tubes and bifurcated tube systems with a diameter smaller than 2 mm aimed at small to smallest supplying systems with capillary dimensions. WST-1 biocompatibility tests ofm polymer extracts show nontoxic characteristics of the adapted polymers after a washing process. Some polymers show shape memory effect (SME).

## 1. Introduction

Functional supplying and disposal systems, namely artificial functional blood vessel systems [1] are of growing interest in tissue engineering in order to create nature-like tissues or even organs. Besides the synthesis of biofunctional materials, their processability with suited 3D processing methods is a key issue to overcome the current limitation of engineering *in vitro* tissues. Different strategies to obtain blood supplying systems are described in the literature: A bilayer scaffold from electrospun, biodegradable PCL/collagen material is described by Ju *et al*. [2] in which the fibers are fabricated around a several mm thick mandrel. A sub millimeter supplying structure has been realized with a sacrificial cotton candy sugar structure which penetrates the surrounding matrix material (PCL or epoxy resin) by Bellan *et al*. [3]. A sophisticated route is described by Dahl *et al*. using a decellularized matrix tube which resulted from a biodegradable template vascularized in the first place with human muscle cell leaving an extracellularized tube ready to be implanted for vessels bigger than 6 mm or to be seeded with human aortic endothelial (HAEC) cells for smaller sized vein implants (3–4 mm). Recently, an increasing interest in rapid prototyping methods has been observed. The applications range from hard segment implants for skull, hip, dental templates and surgical aid tools [4,5] to tissue engineered scaffold structures with highly complex 3D-porous structures of one with the building element [6]. The groups associated with Liska and Stampfl showed that photopolymers are useful materials for rapid prototyping [7,8,9] with tunable mechanical [10] and in part good biocompatible properties [11]. Bens *et al*. [12] described photocurable ethoxylated bisphenol A polyacrylates with non-toxic and flexible characteristics. Baudis *et al*. [13] adapted photoelastomers in digital light processing (DLP) for vascular tissue regeneration with similar mechanical properties as natural blood vessels with dimensions just below 1 MPa for Young’s module and 1 MPa in tensile strength. This is comparable to natural conditions varying in sizes and assignment [14]. The material aspect plays an eminent role in fulfilling the demands of the process: the manifold qualities of a functional blood vessel implant system such as mechanical flexibility and elongation, biocompatibility, cell adherence and non-thrombotic properties and even the geometry of anastomosis [15]. We recently showed that MPP with a bisphenol A ethoxylated diacrylate in reactive dilutions allows sub millimeter 3D-scaffold structuring of polymer-protein hybrid structures with a Young’s modulus of the used polymer bulk material of 22 MPa and an elongation strength of 3 MPa [16,17]. We also demonstrated that photocurable inks can be easily functionalized with carboxygroups without losing their biocompatible and cell adherency ability [18]. Within this study, we demonstrate that a simple design of our investigated oligomers containing photo reactive endgroups, yields photo-crosslinked polymers with different physical but similar chemical properties. These materials fulfill the prerequisite for two different 3D structuring methods, capable of generating bifurcated tube systems similar to a natural blood vessel system in dimensional and mechanical aspects: stereolithography (SL) and two-photon polymerization (MPP). These method processes are able to generate arbitrary 3D structures by laser-induced photocuring of a photosensitive resin. SL normally has a resolution of approximately 5–50 µm, whereas MPP can achieve resolutions below 1 µm. However, for large scale structures, SL is usually preferred, because of its higher process speed.

Firstly, the mechanical characteristic is adapted maintaining the biocompatibility of vessel-like substitute materials. We expect, according to the growing spacer length of the resins in this series a wider polymeric network and therefore a direct effect on the mechanical properties to lower E-module values that fit the initial mentioned mechanical properties of natural blood vessels and are suited for SL processing methods. Secondly, millimeter and sub-millimeter sized artificial capillary and bifurcated supplying modules are processed.

## 2. Results and Discussion

**Figure 1:**
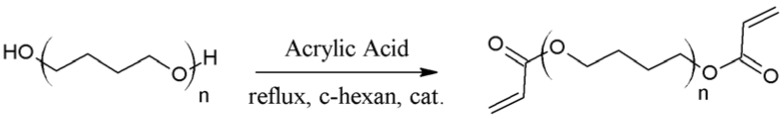
Endgroup functionalization of α,ω-dihydroxy-polytetrahydrofuran (PTHF) by catalytic esterification with acrylic acid analogue to the described method in [19] resulting in PTHF-DAs 2–4.

A homologues series of four α, ω-dihydroxy-polytetrahydrofuran-diacrylates (PTHF-DA) has been investigated. One commercially available PTHF-DA (1) and three PTHF-DAs (2–4) (Figure 1) with higher molecular weights were synthesized according to a procedure described in the literature [19] and characterized with NMR, GPC, DSC and rheology. Curing times were studied under UV-light exposure and FTIR detection.

**Figure 2:**
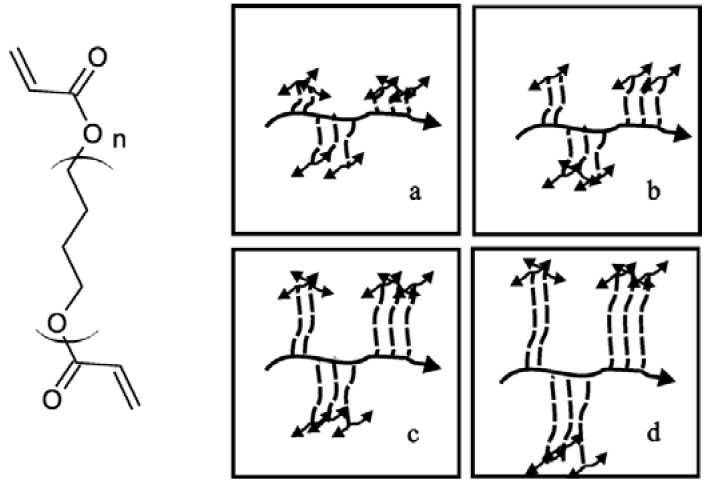
Prepolymer structures with n repetitive PTHF units, terminal acrylate groups (left) and schematic polymer network PTHF-PA 1-4 from the corresponding PTHF-DA 1-4 with increasing numbers of repetitive units (n= 13, 34, 52, 67) from **a**–**d**. Bold arrow: polyacrylate backbone; dashed line: PTHF-spacer unites; double arrows: backbone network connection.

Beside the determination of the E-modulus and the tensile strength of the photo-cured materials (Figure 2), we studied the swelling behavior in aqueous and organic solvents, the crosslinking degree by FTIR and the thermal transitions by DSC. Finally, the processing of small (Ø_inner_ ~2mm) and smallest tube systems (Ø_inner_ ~20 µm) by means of stereolithography and MPP, respectively, is described by using PTHF‑DA 1, which is best processable at room temperature due to its low viscosity. 

### 2.1. Characterization of Prepolymers PTHF-DA 1-4

Table 1 summarizes the characteristic physical parameters of the homologues series of the PTHF-DA 1-4 prepolymers. Within this series the molar masses increase from 1,050 to 4,936 g·mol^−1^ with a polydispersity from 1.67 to 2.36. According to the increasing molar masses an expected increase in viscosity and melting point values is observed. 

**Table 1:** Physical parameter of prepolymers α, ω-polytetrahydrofuranether-diacrylate (PTHF-DA)1-4.

PTHF-DA	M_n_ [g·mol^−1^] [a]	P [a]	n [a]	mp. [°C] [b]	viscosity [mPa·s] [c]
1	1,050	1.67	13	5–12	27
2	2,544	1.99	34	22	151
3	3,856	1.99	52	26–37	314
4	4,936	2.36	67	40.5	932

[a] from GPC, P is polydispersity, n calculated number of repetitive unit in PTHF-DA; [b] from DSC; [c] at 60 °C

#### 2.1.1. FTIR

**Figure 3:**
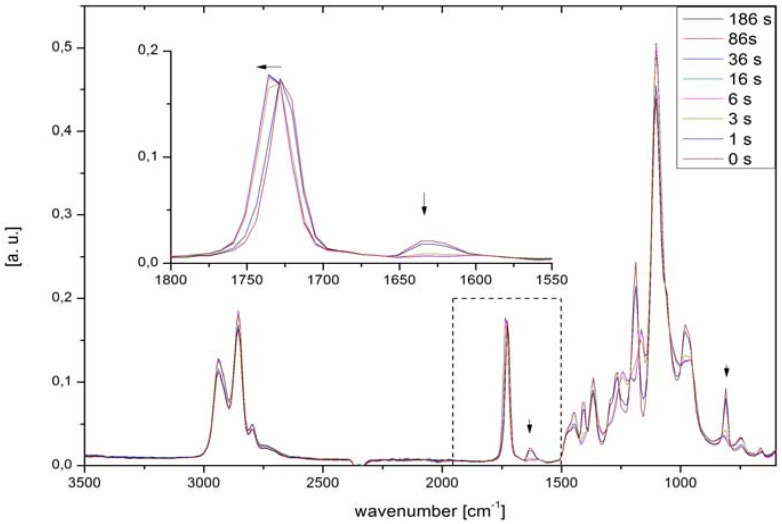
ATR-FTIR spectra of PTHF-DA 1 turning into PTHF-PA 1 recorded after different exposure times to UV light. Fast curing of a 100 µL droplet on FTIR crystal is observed in seconds. Double bond conversion is determined as described in [18].

Figure 3 shows characteristic FTIR spectra recorded during exposure to UV irradiation of PTHF-DA with 0.5% photoinitiator Irgacure^®^ 184. The double bond band at 1,630 cm^−1^ and at 810 cm^−1^ decreases with the curing time. Already after 3 seconds, about 90% conversion, the reactive double bonds have vanished. If the material is cured longer than 6 seconds the double bond band at 1,630 cm^−1^ disappears, which indicates a full conversion of the materials. Interestingly, there is no decrease in curing times within the homologues series, although longer necessary exposure times are expected in blend materials with a higher molar mass but smaller acrylate concentration. 

### 2.2. Characterization of Polymers PTHF-PA 1–4

Physical characterizations of PTHF-PA 1–4 are summarized in Table 2. We found slight water swelling of the polymers with values between 1.3–1.9% independent of the molecular mass of the used prepolymer. A surface energy effect between the examined polymers and water is observed by the contact angle measurements, indicating a growing hydrophobic characteristic with values from 64° to 84° within the homologous series. The decrease of the contact angle for PTHF-PA 4 may be due to coiling effects of the corresponding prepolymer that result in fact to a shorter head to tail distance of the reactive prepolymer and actually could be classified between polymer 2 and 3 regarding the physical behavior. This situation is in good agreement with the DSC and Young’s modulus measurements.

#### 2.2.1. DSC

**Figure 4:**
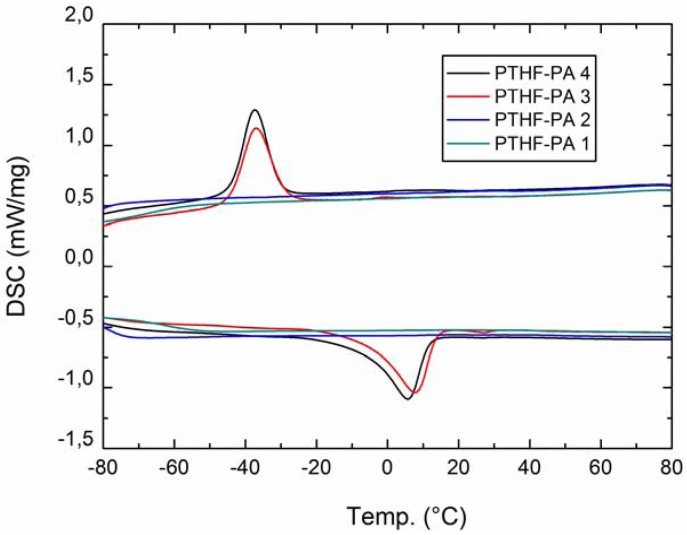
DSC (second run) from PTHF-PA 1-4. Transformation peaks occur only within PTHF-PA 3+4 indicating folding phenomena within the PTHF sequences.

DSC measurements of the homologues series PTHF-PA 1-4 were performed from −80 °C to 80 °C (Figure 4). As expected, the glass transition can be observed weakly at very low temperatures below −60 °C for very amorphous, transparent photocured polymers. However, strong melting transitions are observed at 4–5 °C respectively for PTHF-PA 3 and 4. This characteristic transition for the two polymers with higher molar masses is a known phenomenon caused by the folding and packing of the long chain PTHF network segments. Recrystallization transition was observed for both these polymers in the cooling curves at around 38 °C, above the melting temperature the molecules untangle. However, folded polymers have a smaller head to tail distance and the effective length of the molecules is smaller within the polymeric network, which can be seen in the elasticity measurements, e.g., in a higher E-modulus for PTHF-PA 4 compared to the value of PTHF-PA 3. In addition, a small melting peak occurs sporadically at 26 °C for PTHF-PA 3, which is assignable to the melting point of the corresponding prepolymer PTHF-DA 3 and which originates from not fully cured prepolymers enclosed in the polymeric network.

#### 2.2.2. Mechanical Characterization

The Young’s modulus and tensile strength of bulk material probes PTHF-PA 1–4 were examined and results are shown in Table 2. For the polymer materials PTHF-PA 1–3 a decrease of the Young’s modulus (tensile strength) values from approximately 30 MPa down to 6 MPa is observed. PTHF-PA 4 exhibits a Young’s modulus of 8 MPa. The decrease of Young’s moduli is exxpected because of the increasing molar masses of the prepolymers PTHF-DA 1-4 which leads to a wider polymeric network in their products. Within this dependence, PTHF-PA 4 is an exception. This is explainable due to the polymeric coil structure of the diacrylate 4 having molar masses above 3800 g/mol. The PTHF-DA 3 and 4, own folded and coiled structure elements, result in additional physical nodal points within their polymeric network PTHF-PA 3 and 4 which have more effect on Young’s modulus. This observation is in good agreement with the DSC results where melting and freezing of these structures is apparent. 

**Table 2:** Physical parameter of polymers PTHF-PA 1-4.

PTHF-PA	swelling in H_2_O [%]	Contact angle_adv_ [°]	Contact angle_rec_ [°]	Young’s modulus [MPa]	Bending strength [MPa]	T_c_/ΔH_c_ [°C]/[J/g]	T_m_/ΔH_m_ [°C]/[J/g]	Gelation [%]
1	1.3	64.2 ± 5.8	39.2 ± 1.9	27.5	3.5	-	-	98.2
2	1.9	72.5 ± 3.4	50.1 ± 1.3	10.7	1.8	-	-	97.2
3	1.5	84.8 ± 8.0	42.4 ± 2.6	5.7	1.3	−37.0/39.6	7.2/42.8	97.2
4	1.9	77.5 ± 2.8	44.0 ± 2.2	8.0	1.1	−37.3/39.6	5.0/42.4	98.6

#### 2.2.3. Shape Memory Effect

**Figure 5:**
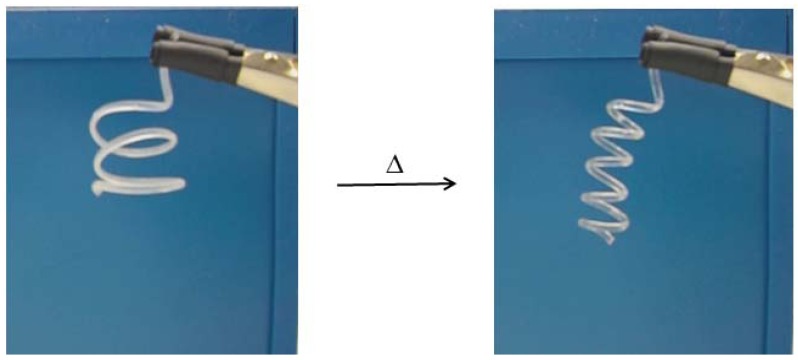
A three winding polymeric spring programmed at −20°C, changes back into its original shape—a five winding spring at room temperature—within 34s. Here, the spring is made fully of PTHF-PA 4. A video of this transition is provided in the additional information.

Interestingly, we found shape memory effect (SME) attributes in the polymer material PTHF-PA 3 and 4 indicated by the melting and crystallisation effects: shown by the above discussed melting behaviour in 2.2.1. Regarding the purpose of processing a medical device, SME is a useful and interesting characteristic. Regarding the difficulties in sewing smallest blood vessels to their natural counterpart, SME offers the possibility of an alternative way of connectivity, such as self-tidying sewing thread as described in [20]. In order to demonstrate the shape memory effect, we photopolymerized a five winding spring made from PTHF-PA 4 and reshaped it at room temperature into a three winding spring. Then, the new photopolymerized shape was stored for one hour at −20 °C. After transformation to room temperature, the polymeric flexible spring changes to its original shape. After keeping the new shape for one hour at −20 °C, the polymeric and flexible spring changes to its original shape at room temperature (Figure 5). A video of the transition can be found in the additional information.

#### 2.2.4. Biocompatibility

**Figure 6:**
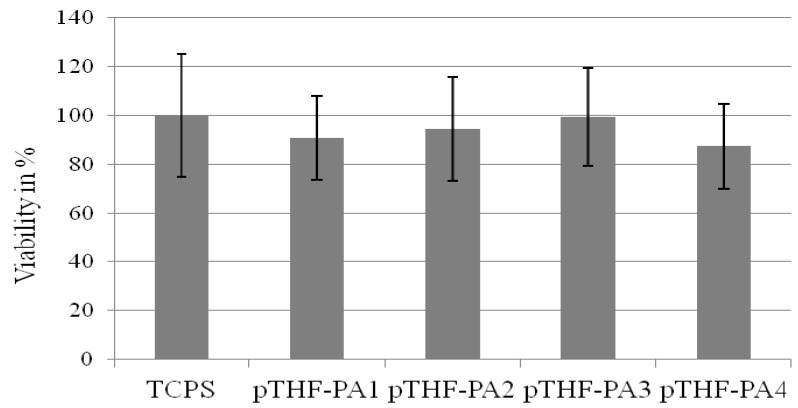
Viability of human fibroblasts on material PTHF-PA 1-4 and control material tissue culture polystyrene (TCPS). WST-1 assay was performed 48 h after seeding. TCPS was taken as a reference (100%). All materials show a cell viability higher than 87%.

Cell viability was measured by a WST-1 assay with human dermal fibroblasts after treatment with extracts from the intensely washed materials PTHF-PA 1-4 and the control material (Figure 6). The gel content was 97–98% (see Table 2) for all materials showing no significance in the WST-1 test. For material PTHF-PA 1, cell viability was 90% ± 10%, lower than for the control substrate extract (defined as 100% ± 25%). Cells treated with extracts of material #2 showed a 5% reduced viability compared to cells treated with extracts from TCPS. In comparison, after treating with TCPS extracts, viability of the cells treated with #3 and #4 receded, for 0.5% (#3) and for 12.5% (#4).

#### 2.2.5. Stereolithography Processing (SL)

**Figure 7:**
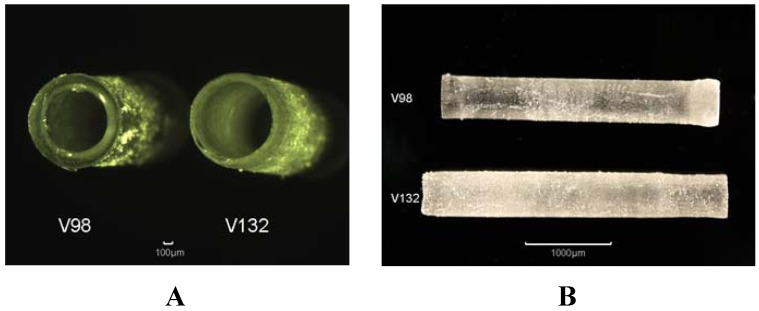
**A**:3D tubes made from PTHF-DA 1 with 0.5% Irgacure-184 and 10% additional crosslinker trimethylolpropane triacrylate (V98, rotation time 40 s, constant velocity in z-direction) and **B**: PTHF-DA 1 (V132, rotation time 60 s, variable velocity in z-direction)

In order to process a cylindrical structure, a pulsed nitrogen laser (wavelength 337 nm, pulse duration 0.5 ns, pulse repetition rate 4 Hz) is focused by a fused silica lense with a focal length of 75 mm to form a focus diameter of nearly 10 µm on the rotating surface in the photopolymer containing vat. The exposure time for the polymer depends on the motion stage rotation speed and the repetition rate for the focused laser spot. With a rotation speed in the range of 100 µm/s and a velocity in the z-direction of 6 µm/s several exposure loops are necessary in order to process one layer with a diameter of 2 mm. The polymerization process is repeated layer by layer after translation of the sample holder in the z-direction. Examples of processed tubes are shown in Figure 7. Because of the high focus depth and in order to prevent a longer polymerization time for the previous layers, a gradient in the z-direction velocity is necessary to realize a nearly constant polymerization procedure for all layers and thus a constant wall thickness of the tube. The component geometry of the polymerized structure can be varied by movement of the motion stages in all directions. 

#### 2.2.6. Multiphoton Polymerization (MPP)

**Figure 8:**
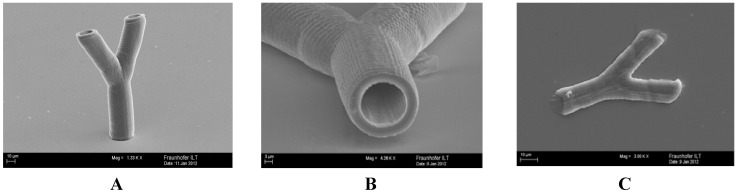
Scanning electron micrographs of branched tubular structure, generated by two-photon polymerization of PTHF-PA 1. The height of the tubular structures is approximately 160 µm (**A**); where the inner diameter and wall thickness is approximately 18 µm and 3 µm (**B**); for smaller wall thicknesses of about 1 µm the tubular structure collapses, due to the elasticity of the material (**C**).

In order to generate 3D vessels from PTHF-PA 1, a mean laser power of 9.8 mW is used. The separation distance of the two adjacent z-layers is 2 µm, and the distance of the xy-plane filling lines is 0.5 µm. Both length scales can be identified on the finalized branched vessels (Figure 8). The inner diameter of the vessel is 18 µm, the wall thickness is 4 µm and is slightly smaller in the branched arms. The height of the branched vessels is 160 µm.

## 3. Experimental Section

### 3.1. Instrumentation

Chemicals: NK-ESTER A-PTMG-65 (Shin-Nakamura, Wakayama, Japan) (PTHF(775)-diacrylate) was kindly supplied by Kowa Europe GmbH. All other chemicals were purchased by Sigma-Aldrich^®^ (St. Louis, MO, USA) and were used without further purification. High-resolution (500 MHz) ^1^H-NMR spectra were recorded using a Unity Inova 500 spectrometer (Varian, Germany). DSC measurements were carried out at a heating rate of 10 K/min using a DSC 204 F1 Phoenix^®^ (Netzsch, Tirschenreuth, Germany). IR-measurements were performed on a Scimitar 2000 FT-IR (Varian, Germany). Rheometric data were obtained using a Rheo Stress RS150 (Haake, Germany), contact angles were measured with DSA 100 S (Krüss, Hamburg, Germany); gel contents were obtained gravimetrically before and after extraction with chloroform after 48 h.

Synthesis: The PTHF-diacrylates (**PTHF (M_n_ ofeduct)-DA**) were synthesised as described in reference [19]. The reaction scheme of the one step endgroup functionalization is shown in Figure 1. α,ω-Dihydroxy-polytetrahydrofuran-diacrylate **PTHF(1400)-DA**: 40 g Poly-(tetrahydrofuran) (M = 1,400 g·mol^−1^) (28.57 mmol), 5.08 g (4.8 mL, 69.3 mmol) acrylic acid, 0.49 g (2.5 mmol) p-toluenesulfonic acid monohydrate and 0.098 g (0.1 mmol)) hydroquinone were dissolved in 600 mL cyclohexane and refluxed in a Dean-Stark apparatus until no further water separation was observed. After addition of further amounts the reaction mixture was treated with potassium carbonate (K_2_CO_3_) with stirring for 3 h at 40 °C and then filtered off. The solvent was evaporated off and washed with 2.5 mM NaOH until the washing solution was colourless. After washing with water until pH neutral, the light yellow, viscous residue was dried under vacuum for 48 h yielding 90% of theoretical value. ^1^H NMR(500 MHz, CDCl_3_, δ): = 1.6 (CH_2_, 78H, brs), 3.4 (-O-CH_2_, 78H, brs), 4.2(CH_2_-Ac, 4H, t), 5.8 (=CH_2_, 2H, d), 6.18 (-CH, 2H, dd), 6.4 (=CH_2_, 2H, d), FTIR(cm^−1^): ν = 813, 1,100, 1,195, 1,369, 1,727, 2,855, 2,929. Following the analogous synthesis with poly(tetrahydrofuran 2,000 g·mol^−1^ and 2,900 g·mol^−1^. **PTHF (2000)**-**DA**: ^1^H NMR(500 MHz, CDCl_3_, δ): = 1.6 (CH_2_, 138H, brs), 3.4 (-O-CH_2_, 138H, brs), 4.2 (CH_2_-Ac, 4H, t), 5.8 (=CH_2_, 2H, d), 6.18 (-CH,4H, dd), 6.4 (=CH_2_, 2H, d), FTIR(cm^−1^): ν = 813, 1,100, 1,195, 1,369, 1,727, 2,855, 2,929, **PTHF(2900)-DA**: ^1^H NMR(500 MHz, CDCl_3_, δ): = 1.6 (CH_2_, 162H, brs), 3.4 (-O-CH_2_, 162H, brs), 4.2(CH_2_-Ac, 4H, t), 5.8 (=CH_2_, 2H, d), 6.18 (-CH, 4H, dd), 6.4 (=CH_2_, 2H, d), FTIR(cm^−1^): ν = 813, 1,100, 1,195, 1,369, 1,727, 2,855, 2,929. 

Biocompatible tests: The proliferation of human dermal fibroblasts was quantitatively evaluated using the water soluble tetrazolium (4-[3-(4-Iodophenyl)-2-(4-nitrophenyl)-2H-5-tetrazolio]-1,3-benzenedisulf-onate) test (WST), which describes the cellular mitochondrial dehydrogenase activity. Metabolism in viable cells produces a reducing equivalent—NADH—that passes its electron to an intermediate electron-transfer reagent, which can reduce WST-1 to the aqueous formazan product. The concentration of the product is proportional to the number of viable cells. Absorbance of the colored formazan product provides a quantitative determination of cell viability. We performed an eluate test where cells do not come into contact with the material directly. The postcured materials were covered with 3 mL endothelial growth medium without supplements and the extraction of the materials was carried out at 37 °C, 5 % CO_2_ for 24 h without shaking. Cell cultures were seeded, at the density of 1 × 104 cells/well, in 96-well tissue culture polystyrene (TCPS) plates and grown for 24 h. The biomaterial extracts were directly added to the cell cultures (200 µL/well), and incubated for 24 h at 37 °C, 5% CO_2_. Medium, incubated on TCPS petri dishes was provided as a positive control. To perform a WST-1 test, 20 µL of WST-1 reagent was added to the cells in medium in each well. The cells were incubated with the WST-1 reagent at 37 °C, 5 % CO_2_ for 1 h. The application of the 1 h incubation period was based on a series of preliminary experiments. After incubation the absorbance was measured with a microplate reader (Tecan, Crailsheim, Germany) with a test wavelength at 450 nm and a reference wavelength at 630 nm. The 630 nm background absorbance was subtracted from the 450 nm measurement.

### 3.2. Processing Setup

**Figure 9:**
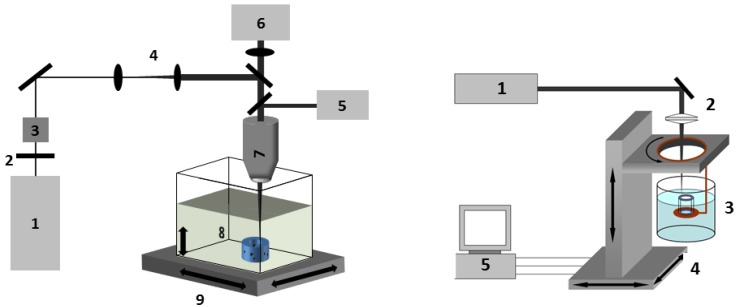
Experimental setup used for multiphoton polymerization (MPP) (left): (1) fs-laser source; (2) λ/2-waveplate; (3) polarizing beam splitter; (4) beam expander; (5) halogen lamp; (6) CCD-camera;(7) objective; (8) material bath; (9) 3D-axis handling. SL (right): (1) nitrogen laser; (2) focus lense; (3) photo polymer bath; (4) translation stage/rotation stage; (5) motion controller.

Multiphoton polymerisation (MPP): The experimental setup for two photon polymerization was described elsewhere [16]. For this study, a Ti:Sa laser system is used which emits pulses with a repetition rate of 80 MHz at a wavelength of 690 nm. The laser beam is directed through a microscope objective (20×, NA 0.5, Olympus, Hamburg, Germany), slightly overfilling its back aperture. Due to practical reasons all power measurements are performed before the laser beam enters the objective. The transmission rate of the objective is determined by measuring the mean laser power before and after the objective and is included in all power values described in this study. A small amount of the photosensitive resin (for TPP 3% Irgacure-184 is used) is placed on a glass slide and treated with 3-(trimethoxysilyl)propylmethacrylate (Sigma-Aldrich, St. Louis, MO, USA) according to the procedure provided by the supplier in order to improve matrix adhesion to the glass surface. In order to generate a smooth resin surface the sample was covered with a coverslip, separated by 170 µm thick glass spacers.

After crosslinking the samples are rinsed for 2–3 min in 70% ethanol for the removal of non-crosslinked resin, dried at room temperature and sputter-coated with gold prior to SEM analysis.

## 4. Conclusions

Synthesis of a homologous series of photocurable α,ω-PTHF-diacrylate (PTHD-DA) oligomers was achieved with high yields. These materials are suited for stereolithography and photopolymerization and show important properties in tissue engineering applications such as biocompatibility, mechanical flexibility and shape memory effect. UV- and MPP stereolithography are suitable to cure PTHD-DA to process blood vessel-like supporting structures from mm down to sub µm dimension. The combination of these two laser supported processing methods appears to be a promising way to structure artificial blood vessel supplying devices that are for example needed for tissue engineering and will be a topic of further investigation. The polymeric materials show biocompatible abilities and have mechanical attributes comparable to those of blood vessel capillaries. Future studies will also investigate biofunctionalisation of the presented materials and cell adhesion studies.

## Supplementary Files

Supplementary File 1 FLV-Document (FLV, 2160 KB)
